# TSFN: A Novel Malicious Traffic Classification Method Using BERT and LSTM

**DOI:** 10.3390/e25050821

**Published:** 2023-05-19

**Authors:** Zhaolei Shi, Nurbol Luktarhan, Yangyang Song, Huixin Yin

**Affiliations:** College of Information Science and Engineering, Xinjiang University, Urumqi 830046, China; shizhaolei@stu.xju.edu.cn (Z.S.); song_yy@stu.xju.edu.cn (Y.S.); 107552103573@stu.xju.edu.cn (H.Y.)

**Keywords:** malicious traffic classification, long short-term memory, pre-training, bidirectional encoder representations from transformers

## Abstract

Traffic classification is the first step in network anomaly detection and is essential to network security. However, existing malicious traffic classification methods have several limitations; for example, statistical-based methods are vulnerable to hand-designed features, and deep learning-based methods are vulnerable to the balance and adequacy of data sets. In addition, the existing BERT-based malicious traffic classification methods only focus on the global features of traffic and ignore the time-series features of traffic. To address these problems, we propose a BERT-based Time-Series Feature Network (TSFN) model in this paper. The first is a Packet encoder module built by the BERT model, which completes the capture of global features of the traffic using the attention mechanism. The second is a temporal feature extraction module built by the LSTM model, which captures the time-series features of the traffic. Then, the global and time-series features of the malicious traffic are incorporated together as the final feature representation, which can better represent the malicious traffic. The experimental results show that the proposed approach can effectively improve the accuracy of malicious traffic classification on the publicly available USTC-TFC dataset, reaching an F1 value of 99.50%. This shows that the time-series features in malicious traffic can help improve the accuracy of malicious traffic classification.

## 1. Introduction

Traffic classification is an essential technology in network security and network management [1]. Traffic classification techniques can classify network traffic into specific application types and serve an important role in tasks related to the quality of service assurance, quality of experience (QoE), intrusion detection, network monitoring, and network visibility [2]. With the skyrocketing advancement of Internet technology, the exponential growth of network traffic data volume, and the increasing demand for privacy and confidentiality from more and more users, traffic encryption techniques have been widely used and significantly developed. However, malicious traffic using encryption disguise technology is also increasingly rampant, bringing serious challenges to traffic classification methods. Encrypted traffic not only guarantees the privacy and anonymity of most users, but also poses a significant challenge to cyberspace security [3]. Some unscrupulous criminals employ advanced traffic encryption technologies to accomplish criminal activities, which not only seriously jeopardizes the stability of cyberspace, but also seriously impacts national security. Network traffic is an essential vehicle for Internet communication, and traffic data contain a great deal of valuable information. Therefore, an increasing amount of researchers are devoting themselves to the research of network traffic [4].

Earlier classification approaches have yet to be well-suited for encrypted traffic [5]. These methods detect emerging traffic by analyzing previous traffic data and extracting feature strings as traffic fingerprints to perform matching operations. Deep Packet Inspection (DPI) [6], for example, cannot be applied to encrypted traffic because this approach uses plaintext traffic data for its work [7]. Many researchers have tried to use machine learning approaches for network traffic classification to overcome this problem. One of these methods is the statistics-based approach. The primary principle is that different applications generate traffic with different features. For example, the traffic of web applications commonly exhibits many bytes generated in a short time, and the traffic of Voice over Internet Protocol (VoIP) applications usually exhibits a small number of bytes transmitted steadily in a short time [3]. The researcher selects feature data that reflect the features of the class and then chooses some machine learning model for training. The trained model can classify new traffic [5]. Statistical-based methods for classifying network traffic have the advantages of being lightweight and being applied to encrypted traffic. However, the performance of the statistical-based approach is easily affected by the quality of the feature design, and the method does not generalize well.

With the outstanding performance of deep learning in natural language processing and image recognition, researchers have tried to apply deep learning in network traffic classification. Deep learning methods have the advantage of automatically extracting complex features. However, when deep learning-based methods perform network traffic classification, it is susceptible to learning biased features due to unbalanced datasets [4]. In addition, the amount and distribution of labeled data can also impact the model’s feature learning. In recent years, a breakthrough in natural language processing has occurred with the emergence of Bidirectional Encoder Representation from Transformers (BERT) pre-training models, and remarkable results have been achieved in several fields [8]. By pre-training on large amounts of unlabeled data, BERT learns unbiased generic data features and can easily use these features in downstream tasks. Consequently, researchers have attempted to utilize BERT models for network traffic classification. However, some currently existing BERT models ignore the time-series level features in the network traffic and use only the global features of the traffic for classification prediction.

This paper proposes a novel model called the time-series feature network model (TSFN), to improve the performance of malicious traffic classification. To overcome the deficiencies of existing malicious traffic classification methods, the work in this paper includes first extracting the global features of malicious traffic using the BERT model and then using the Long Short-Term Memory networks (LSTM) model to capture the time-series features in malicious traffic and combine the two into a final malicious traffic feature representation, which can better represent malicious traffic and thus improve the performance of malicious traffic classification. Compared with the existing BERT model, the accuracy of our proposed method for malicious traffic classification on the USTC-TFC dataset is improved by 0.2% to 99.49%; and the identification performance on three classes, Virut, Outlook and Neris, is more obviously enhanced, with F1 values improving by 4%, 1.3% and 1.3%, respectively. The main contribution of this paper is to propose a malicious traffic classification model based on BERT and LSTM, which is a structure that can capture both global and time-series features to obtain a better representation of malicious traffic features. In this paper, we conduct numerous experiments on the UST-TFC dataset, and the results indicate that our approach is reasonable and effective.

## 2. Related Work

### 2.1. Malicious Traffic Classification

Malicious traffic refers to network traffic that is carefully designed to attack a network, system, or application. Hackers usually use malicious traffic to carry out various network attacks, such as denial-of-service attacks, data theft, and malware distribution, thus causing severe threats and losses to the economic interests and information security of enterprises, governments, and users. In recent years, malicious software commonly employs traffic encryption techniques in order to circumvent the detection of firewalls and antivirus software. For example, many Trojans use Transport Layer Security (TLS) protocols to encrypt network traffic, thus making some malicious traffic detection methods ineffective in detecting and intercepting it. Almost all malware currently uses traffic encryption techniques, which pose a great challenge to malicious traffic detection. Therefore, the research and development of effective malicious traffic detection and defense techniques are of critical importance for cyberspace security.

In this chapter, we summarize the vital traffic identification approaches. Based on the characteristics of the techniques, the existing methods are grouped into the below four types: port-based, constructive fingerprinting techniques, statistical methods, and deep learning models [9].

Port-based: In the early days of the Internet, traffic classification was a relatively simple task, usually identified by the port number at the transport layer, because all applications were assigned a public, fixed port number [3]. Researchers only need to parse the packet headers of network traffic data, extract the port numbers, and then query the port mapping table to determine the class to which the traffic belongs [10]. However, with the swift advancement of the Internet, modern programs popularly employ technologies such as random port policies [11], masquerading port techniques, and Network Address Translation protocols (NAT), which make port-based classification methods increasingly inappropriate for modern network environments. In contrast, our method does not use any port numbers for classification.

Fingerprint Construction: There are mainly two types of methods based on constructing fingerprinting techniques, one represented by Deep Packet Inspection [6] and the other by FlowPrint [7]. DPI methods analyze past attack traffic data and extract specific strings as traffic fingerprints by building a library of attack traffic fingerprints and performing regular pattern matching on newly emerged The DPI approach analyzes past attack traffic data and extracts specific strings as traffic fingerprints, and identifies them by building a library of attack traffic fingerprints and performing regular expression matching on newly emerged traffic [5]. FlowPrint [7] uses unencrypted information from encrypted traffic, such as credentials, device, and time characteristics, for identification. These approaches are highly reliant on plaintext information. In contrast, our approach does not rely on plaintext information for classification.

Statistical Methods: The main principle of the statistics-based approach is that different kinds of applications generate distinctive traffic features. Researchers select feature data that reflect these characteristics and choose appropriate machine learning models for training. After training, these models can identify and classify new traffic. For instance, AppScanner [12] utilizes the packet size for traffic classification, while BIND [13] utilizes temporal-related statistical features for classification. Statistical-based approaches for network traffic classification do not require byte-by-byte traffic inspection and thus have the advantage of being lightweight. Furthermore, since these methods use only statistical information about the traffic, they effectively classify encrypted traffic. However, these methods require specially designed features, which makes them time-consuming, costly, and laborious [4]. In contrast, our approach does not need to depend on human-designed features for classification.

Deep Learning Model: Because deep learning has shown excellent performance in several domains, researchers have started using deep learning methods for traffic classification. These deep learning-based methods can automatically extract features from raw traffic and attain high classification accuracy. DF [14] and FS-NET [15] utilize deep learning to auto-capture representations from raw packet size sequences, one using Convolutional Neural Network (CNN) and one Recurrent Neural Network (RNN). Deeppacket [16], TSCRNN [17], and wang’s approach [5] utilize deep learning to classify traffic directly using raw traffic. Jay [18] proposed a malicious traffic classification method based on convolutional neural networks and bi-directional long- and short-term memory networks, which achieved 86% of the F1 values on the UNSW-NB15 dataset. HCRNNIDS [19] employs convolutional and recurrent neural networks to classify malicious traffic on the CIC-IDS2018 dataset, achieving an accuracy of 97.75%. Chin-Shiuh [20] proposes a convolutional neural network-based method for classifying malicious traffic and achieves higher classification accuracy through deep learning techniques. The method achieves 99.79% accuracy on the CIC-IDS2017 dataset and 99.8% in identifying unknown attacks on the CICIDDoS2019 dataset. However, existing deep learning-based approaches rely on a lot of labeled data, and the imbalance of the dataset can significantly impact the classifier’s performance [4]. In contrast, our approach only relies on a few labeled datasets for classification.

### 2.2. Pre-Training Models

Recently, pre-trained models have excelled in several domains. These models achieve task-specific optimization by learning a generic language representation on a large-scale corpus and then fine-tuning it [5]. Despite the lack of obvious semantics of packet payloads in encrypted traffic, Sengupta [21] used the randomness differences between different encrypted traffic to classify traffic, showing that encrypted traffic is not entirely random but has underlying patterns. PERT [22] was the first attempt to introduce a BERT model into network traffic classification. However, the model was not designed to represent encrypted traffic and pre-training tasks, dramatically limiting its generalization ability. In contrast, ET-BERT [4] designed two pre-training tasks. pre-trained on a massive amount of unlabeled traffic data and then fine-tuned with a small amount of task-specific labeled data to achieve excellent performance on multiple tasks. For example, on the USTC-TFC dataset, ET-BERT achieved an F1 score of 99.39% on the malicious traffic classification task.

## 3. Methodology

### 3.1. Model Architecture

**Figure 1:**
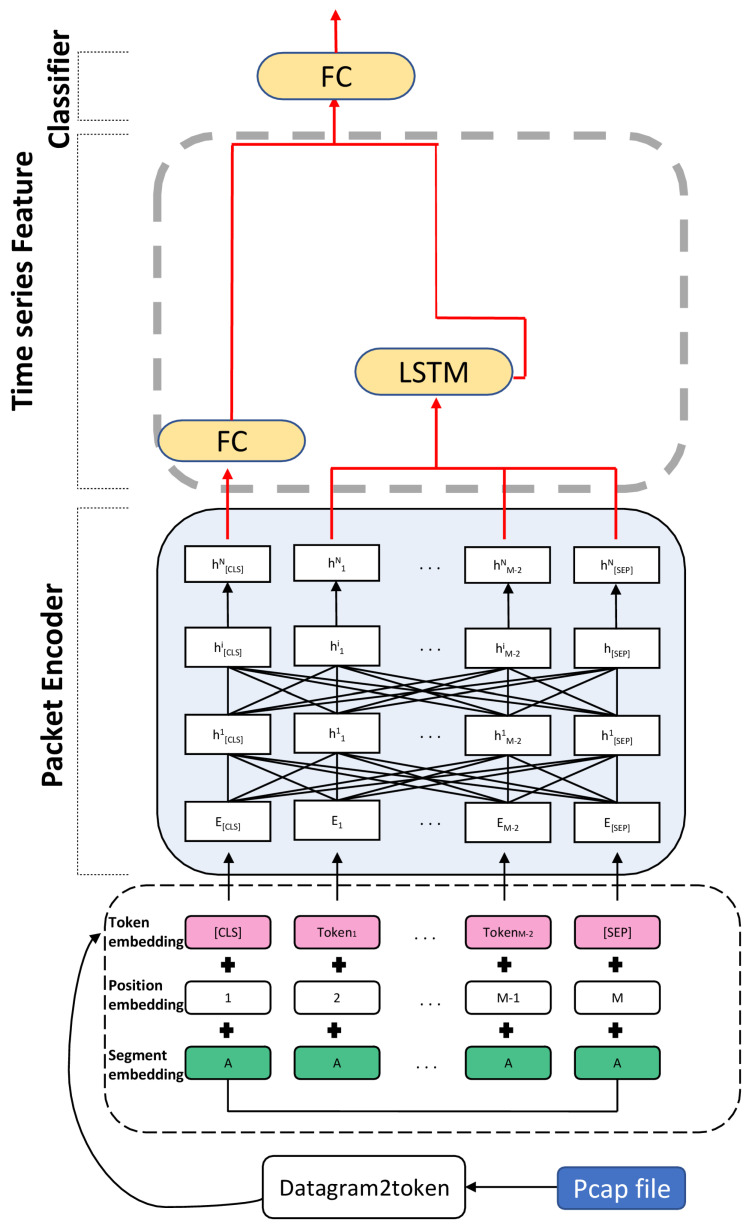
Time-series Feature Network model Structure.

In recent years, approaches combining BERT and LSTM have performed excellently in natural language processing, such as sarcasm detection [23]. Inspired by these studies, we propose a BERT-based time-series feature network model in this paper. The structure of the model is shown in Figure 1, and the model includes three components: a packet-level encoder module, a time-series feature extraction module, and a classifier.

The packet encoder module is constructed using the ET-BERT model [4], which uses a self-attentive mechanism to extract global features from malicious traffic. A token [CLS] is added at the beginning of each sequence; this token does not contain apparent semantic information compared to other tokens. Under the self-attentive mechanism, token [CLS] can more equally integrate the information of each token in the traffic sequence. Thus, the final hidden layer state of token [CLS] in the module better represents the whole traffic sequence and can be used as a global feature to represent malicious traffic. The time-series feature module is constructed using the LSTM model, and its input is the final hidden layer state of the tokens except for token [CLS]. This module can extract the implied time-series features in the traffic sequence through the operation. Finally, the global and temporal features of the traffic sequence are connected as the final traffic feature representation. We input this feature into a classifier composed of fully connected layers to obtain prediction results.

This model is designed and implemented to increase malicious traffic identification accuracy. Through feature extraction of traffic data, this model can effectively distinguish and classify malicious traffic, thus providing a reliable guarantee for network security.

A complete token representation is obtained by adding token embedding, position embedding, and segment embedding.

Token embedding: Each unit of the input sequence is treated as a token, and each sequence is added with a token [CLS] at the beginning and a token [SEP] at the end. When the input length is less than the model length requirement, the token [PAD] is filled at the end of the sequence. The truncation operation is performed when the input sequence length exceeds the model length requirement.

Position embedding: Since the traffic data are transmitted in chronological order, we use position embedding to ensure that the time-series features in the traffic are not lost, enabling the model to identify the time-series relationships between different tokens.

Segment position: This segment is mainly used in pre-training, and this paper does not involve the work of BERT pre-training.

### 3.2. Datasets

A reliable open-source dataset is an essential foundation for researching deep learning algorithms. Using open-source datasets helps to ensure the credibility of research results and facilitates the reproduction and comparison of research work by other researchers. In the field of malicious traffic classification, there are several publicly available datasets, such as USTC-TFC [5], KDD-Cup99 [24], and UNSW-NB15 [25]. However, several datasets suffer from some shortcomings. For example, the KDD-Cup99 dataset contains much redundant and duplicate traffic, while the UNSW-NB15 dataset contains outdated and unreliable traffic. Compared with the first two datasets, the traffic samples in the USTC-TFC dataset do not have too many redundant and duplicate traffic samples. The traffic in this dataset is encrypted using a Transport Layer Security (TLS) protocol, which more closely matches the real network situation. Therefore, we choose the USTC-TFC dataset as the experimental dataset.USTC-TFC includes ten types of malicious traffic and ten types of normal traffic. The malicious traffic in this dataset was collected from CTU University researchers in natural network environments. Traffic that is small in file size and belongs to the same application was merged, and traffic that is large in file size was Truncated. The USTC-TFC dataset contains traffic classes, as shown in Table 1 below. Its traffic is pcap format files with a total size of 3.71 GB. because of its closeness to the natural network environment, this dataset is widely used in the research of malicious traffic identification and classification algorithms.

### 3.3. Data Preprocess

To reduce redundant and interfering information in the traffic, we use the Datagram2Token tool [4] for pre-processing work. The first step is to perform data cleaning to remove traffic unrelated to the transmission content, such as Address Resolution Protocol (ARP) and Dynamic Host Configuration Protocol (DHCP) packets. Because IP and port numbers introduce interfering information, we remove Ethernet headers, IP packet headers, and protocol port numbers for Transmission Control Protocol (TCP) [16]. The hexadecimal traffic sequence is then bi-gram encoded such that each unit is due to two adjacent bytes. Finally, the dataset is divided into a training, validation, and test dataset according to the ratio of 8:1:1. The form of the pre-processed data is shown in Table 2.

## 4. Experiment

### 4.1. Experiment Setting

All experiments in this paper are implemented using Pytorch and universal encoder representations (UERs) [26], running on the Ubuntu operating system with a 3090 GPU with 24 GB memory size. We train four epochs using the AdamW optimizer [27], where the learning rate is 2 × $10^{-5}$. The batch size is set to 32, and the dropout rate is set to 0.5. Our loss function consists of a log-likelihood loss function and a mean-squared error loss function, which is used for model training.

### 4.2. Evaluation Metrics

In this paper, we adopt four classical evaluation metrics, which are Accuracy (AC), Precision (PR), Recall (RC), and F1-score. TP is the number of positive samples correctly classified, FP is the number of negative samples incorrectly classified, TN is the number of negative samples correctly classified, and FN is the number of positive samples incorrectly classified. When evaluating multi-class classification methods, both macro-average [28] and micro-average are often utilized. In this paper, the macro-average approach is adopted because it achieves the objective of avoiding biased results due to imbalance between categories by averaging the metric values of all classes directly.

(1) $$\;\mathrm{Accuracy}\;=\frac{\mathrm{TP}+\mathrm{TN}}{\mathrm{TP}+\mathrm{TN}+\mathrm{FP}+\mathrm{FN}}$$

(2) $$\;\mathrm{Precision}\;=\frac{\mathrm{TP}}{\mathrm{TP}+\mathrm{FP}}$$

(3) $$\;\mathrm{Recall}\;=\frac{\mathrm{TP}}{\mathrm{TP}+\mathrm{TN}}$$

(4) $$F1\text{-}\mathrm{score}\;=\frac{2*\;\mathrm{Precision}\;*\;\mathrm{Recall}\;}{\;\mathrm{Precision}\;+\;\mathrm{Recall}\;}$$

### 4.3. Effect of Different Network Traffic Representations

**Figure 2:**
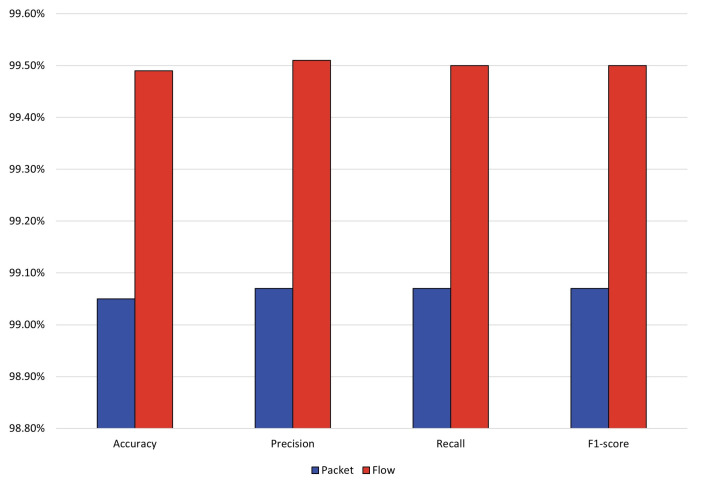
Performance of the model under different network traffic representations.

Network traffic can be split in various ways, and different splits can present very different representations. Researchers need to choose the appropriate network traffic representation to meet practical needs. This subsection selects the most widely used packet-level and flow-level network traffic representations as objects of study. We performed the malicious traffic classification task on the flow-level and packet-level USTC-TFC datasets, respectively, and the experimental results are shown in Figure 2 below.

As can be seen from the above table, the model achieves excellent performance for both packet-level and stream-level network traffic representations, with all four evaluation metrics reaching above 99%. It can be seen that the flow level outperforms the packet level. The accuracy of the model under the flow level exceeds that of the packet level by 0.44%. An intuitive explanation for this result is that flow-level malicious traffic is more conducive for the model to learn the best traffic characteristics. Therefore, the flow level is the most suitable representation for this model. The BERT-based time-series feature model can learn better traffic features from the flow-level form of malicious traffic. The model performs outstandingly in the form of flow-level malware traffic representation and has a remarkably high F1 value of 99.50% on the malicious traffic classification task. Based on the above analysis, this paper adopts the flow-level network traffic representation form for the subsequent research work.

### 4.4. Effect of Sequence Length and the Numbers of Num_Layers

**Figure 3:**
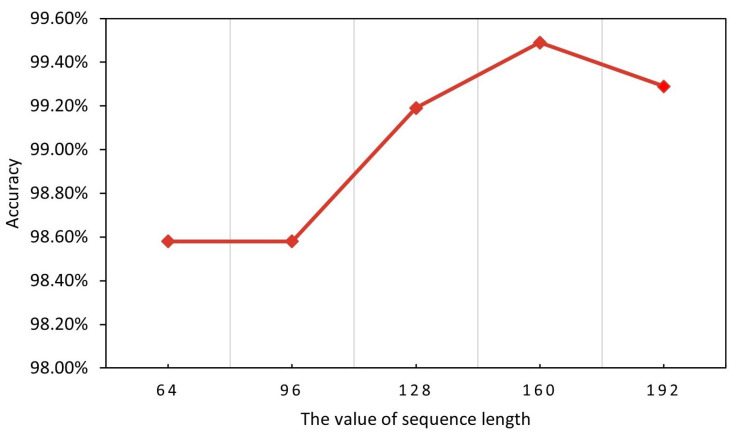
Effect of different sequence lengths (accuracy).

Figure 3 shows the effect of different sequence lengths on the performance of our model. Sequence length is a critical parameter in BERT model training, which directly affects the performance and training efficiency of the model. Sequence length refers to the number of tokens processed by the model. When the input sequence length is smaller than the set sequence length, the model will perform a padding operation; otherwise, it will perform a truncation operation. The larger the sequence length, the more likely it is to introduce meaningless padding tokens [PAD]. The smaller the sequence length, the easier it is to lose meaningful traffic data information. A smaller value will result in losing more traffic information. Therefore, we should choose the most appropriate sequence length to maximize the model’s performance. In this subsection, sequence length is chosen as the object of study to investigate the effect of different sequence lengths on the model performance. The results show that the model’s performance increases and decreases as the sequence length increases. The accuracy of malicious traffic identification reaches the highest rate of 99.49% when the sequence length is 160. Based on the above analysis, we use the sequence length of 160 for the subsequent research work.

**Figure 4:**
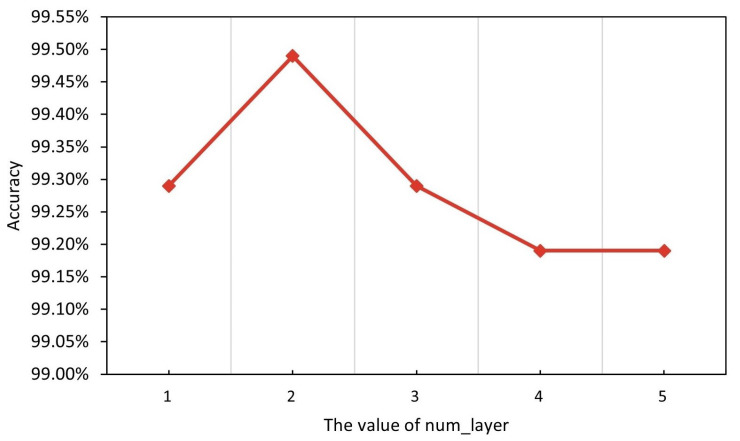
Effect of different num_layer (accuracy).

Figure 4 shows the effect of different LSTM layers on the performance of our model. Num_layer is an essential parameter for LSTM models. Increasing the number of layers of the LSTM can increase the complexity of the model, thus enhancing its fitting ability and, consequently, the accuracy of the model prediction. Each layer of the LSTM can learn and capture information at different time steps, so increasing the number of layers may increase the model’s ability to model time series features. However, when there are too many layers in the LSTM, the model may overfit the training data, resulting in poor performance on unseen data. Too many layers may lead to an overly complex model; thus, the model will over-fit the details of the training data and lose its generalization ability. Therefore, with appropriate layers, the model can better balance the fitting ability and generalization ability to achieve the best accuracy rate. The appropriate number of LSTM layers depends on the specific dataset and task.

The experimental results demonstrate that the model reaches the highest accuracy rate of 99.49% in the malicious traffic classification task when the number of layers is 2. Based on the above analysis, we adopt the value of Num_layer as 2 for the subsequent research work.

### 4.5. Comparison with Different Methods

#### 4.5.1. The Benchmark Methods

In this chapter, 13 existing methods in the field of traffic classification are selected as benchmark methods to compare with our proposed algorithms. These methods are classified as constructed fingerprint-based, statistical feature-based, deep learning-based, and BERT model-based. We have analyzed our methods by comparing them. These analyses will help us to understand the proposed methods better. The benchmark methods are shown in the Table 3.

#### 4.5.2. Experimental Result

The experimental results are shown in Table 4, which demonstrates the experimental results of our proposed model and the existing method on the USTC-TFC dataset. In the malicious traffic classification task, our method outperforms all baseline models regarding accuracy, precision, recall, and F1 values. Our model outshines accuracy, precision, recall, and F1 values with 99.49%, 99.51%, 99.50%, and 99.50%, respectively. Compared with the ET-BERT method using only the BERT model, our method improves 0.2%, 0.21%, 0.2%, and 0.2% in these four metrics, respectively. Compared with wang’s method that proposes the USTC-TFC dataset, our method improves 0.32%, 0.31%, 0.27%, and 0.29%, respectively. The BFCN method, composed of BERT and CNN, fuses global and local features of traffic as the final traffic feature representation. Compared with the BFCN method, the proposed method in this paper improves by 0.1% in terms of accuracy rate. The experimental comparison results demonstrate that combining global traffic features and temporal features of malicious traffic can better represent malicious traffic.

Table 5 demonstrates the identification performance in each class of our method compared with ET-BERT and wang’s method, which proposed the USTC-TFC dataset. As seen from the table, although ET-BERT and wang’s methods have excellent performance, they still have shortcomings in individual categories.

Precision refers to the ratio of the number of samples correctly predicted by the model as positive samples to the total number predicted as positive samples. This paper provides a visual comparison of the accuracy of the three methods for each class identification by the following figure. If the prediction class has a larger proportion of incorrect prediction results, the precision of the prediction class will be lower.

**Figure 5:**
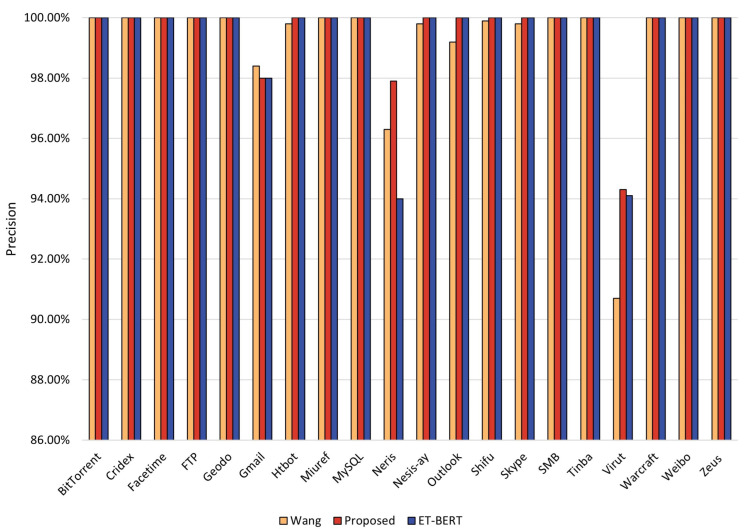
Precision rate of each class in the three methods for malicious traffic classification.

Figure 5 demonstrates the precision of our proposed method and ET-BERT, wang’s method on each class. From the figure, we can find that all three methods have good precision. However, the precision of the ET-BERT method using only the BERT model is only 96.3% and 90.7%. In contrast, our proposed method achieves 97.9% and 94.3% precision for these two categories. Among all 20 classes, our proposed model improves the precision of 7 classes, with 1.6% and 3.3% improvement for the Neris and Virut classes. The classification precision of wang’s method for the Neris and Virut classes is only 94% and 94.1% for the Neris and Virut classes. In comparison, the precision of our proposed method improves by 3.9% and 0.2% for these two classes, reaching 97.9% and 94.3%, respectively.

**Figure 6:**
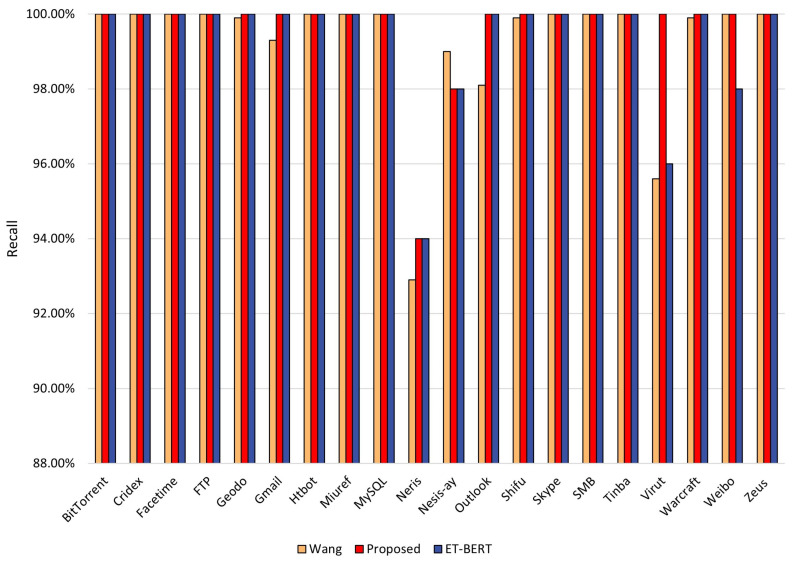
Recall rate of each class in the three methods for malicious traffic classification.

The recall is the ratio of the number of samples correctly predicted by the model as positive samples to the total number of actual positive samples. Figure 6 demonstrates the recalls of the three methods for each class identification. Overall, all three methods have excellent recalls. However, the recall of the ET-BERT method using only the BERT model for Virut and Outlook classes is only 95.6% and 98.1%. In contrast, our proposed method achieves 100% recall for both classes. The recall of our proposed method improved in 7 out of 20 categories, with 4.4% and 1.9% improvement for the Virut and Outlook categories, respectively. The recall of wang’s method is 96% and 98% for the Virut and Weibo categories, respectively. Our proposed method achieves 100% recall for both categories, improving 4% and 2%, respectively.

**Figure 7:**
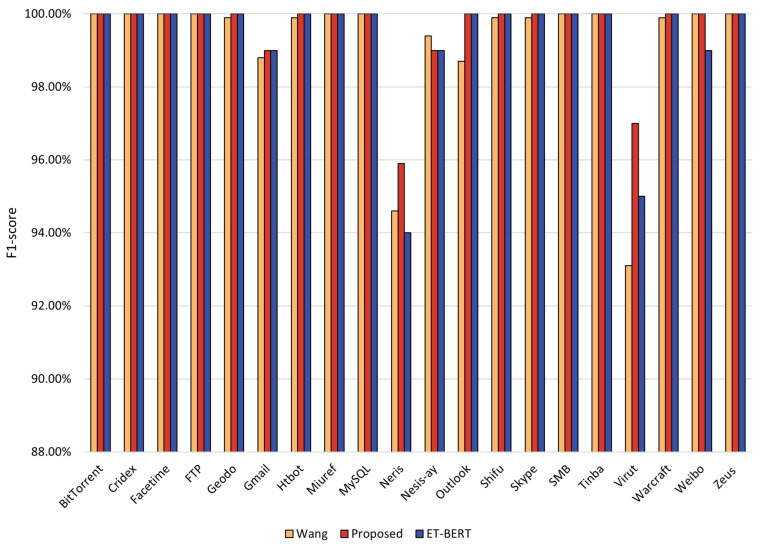
F1-score rate of each class in the three methods for malicious traffic classification.

The F1 score is the summed average of precision and recall, an essential metric for evaluating the model’s performance. Figure 7 visualizes the F1 values for each class of the three methods. In general, all three methods have excellent F1 values. However, for the three classes of Neris, Outlook, and Virut, the recall of the ET-BERT method using only the BERT mode is only 94.6%, 98.7%, and 93.1%. In contrast, our method achieved F1 values of 95.9%, 100%, and 97.1% for these three categories. Among all 20 categories, our method improved F1 scores on 9 categories, including 1.1%, 1.9%, and 4.4% for the Neris, Outlook, and Virut categories. Notably, wang’s method has only 94%, 95% and 99% F1 values for the Neris, Virut and Weibo categories. In comparison, our method improves the F1 scores for these two classes by 1.9%, 2.1%, and 1%, respectively.

In conclusion, our proposed method performs outstandingly well and can accurately classify malicious traffic. Existing methods are affected by the long and information-rich sequences of malicious traffic, resulting in poor classification results, while our proposed method can effectively address this problem. Our model consists of BERT and LSTM models, using BERT to extract global features of malicious traffic and LSTM to extract temporal features of traffic and fusing the two into a final traffic feature representation for malicious traffic identification. The experimental results validate the rationality and effectiveness of our idea.

## 5. Conclusions

In this paper, to improve the accuracy of malicious traffic identification, we propose a novel malicious traffic classification model composed of BERT and LSTM. Our approach can capture the global and time-series features of malicious traffic, fuse these two feature vectors as the final feature representation of the malicious traffic, and finally feed it into the classifier to predict the traffic class. The experimental results demonstrate that our method reaches 99.49% accuracy when performing the malicious traffic classification task on the USTC-TFC dataset, which is a 0.2% improvement over the baseline model. Compared with the existing approaches, our method more fully exploits the features of malicious traffic, using the global features and the time-series features of malicious traffic. Despite the advantages of our approach, it has certain limitations, such as that we do not take into account malware traffic on mobile phones. In future research, we plan to identify more malicious traffic.

## Figures and Tables

**Table 1:** Dataset details.

Traffic Type	Application
Malicious traffic	Htbot, CridexNeris, Nsis-ay, Shifu, Virut, Zeus, Tinba, Miuref, Geodo
Normal traffic	Outlook, BitTorrent, FTP, Warcraft, MySQL, Skype, Facetime, SMB, Weibo, Gmail

**Table 2:** The pre-processed data.

Label	Content
0	021a 1ac5 c502 0200 0000 0002 021a 1ac5 c501 0100 0000 0008 0800 0045 4500 0000 0091 9134 3419 1940 4000 0020 2006 0653 53af af01 0101 01be be9a 9a01 0102 0212 1202 02aa aaba ba01 01bb bbbe be57 57d1 d122 22c8 c853 53cd cd9c 9c80 8018 189e 9e60 60c3 c3e3 e300 0000 0001 0101 0108 080a 0a11 11f5 f594 94b9 b923 2325 2537
1	021a 1ac5 c502 0200 0000 0002 021a 1ac5 c501 0100 0000 0008 0800 0045 4500 0001 0112 1201 016e 6e40 4000 0020 2011 1198 98ac ac01 0101 015e 5e67 6701 0102 025f 5f57 5740 4013 1340 4013 1300 00fe fe1f 1f34 3490 9068 688d 8da2 a257 5732 32af af27 27e4 e49a 9aa8 a8f4 f444 4400 0000 0002 0228 28f5 f527 2773 7300 0000 0000
2	021a 1ac5 c502 0200 0000 0002 021a 1ac5 c501 0100 0000 0008 0800 0045 4500 0005 05b1 b10d 0ddf df40 4000 0020 2006 0617 175c 5c01 0101 015b 5b9f 9f01 0102 02d2 d26a 6ad7 d726 2601 01bb bbb7 b755 5520 2010 10d1 d186 8615 15fd fd80 8018 189e 9e60 6033 33d4 d400 0000 0001 0101 0108 080a 0a11 11f7 f71e 1e54 5423 2326 2638
3	021a 1ac5 c501 0100 0000 0002 021a 1ac5 c502 0200 0000 0008 0800 0045 4500 0000 005e 5e28 2858 5840 4000 0020 2006 06fe fe8c 8c01 0102 02b2 b259 5901 0101 017f 7f59 5900 0015 15b7 b719 19e7 e778 78b4 b400 00e1 e1bd bd05 05e2 e280 8018 1843 43e0 e033 337e 7e00 0000 0001 0101 0108 080a 0ad7 d7f9 f9b9 b95f 5fc6 c671 7188

**Table 3:** The Benchmark Method.

Type	Method
Fingerprint construction approach	FlowPrint [7]
Statistical feature approached	AppScanner [12], CUMUL [29], BIND [13], k-fingerprinting (K-fp) [30]
Deep learning approaches	Deep Fingerprinting (DF) [14], FS-Net [15], GraphDApp [31], TSCRNN [17], Deeppacket [16], wang [5]
pre-training approaches	PERT [22], ET-BERT (flow) [4], ET-BERT (packet) [4], BFCN [9]

**Table 4:** Comparison results on malicious traffic classification [4].

Method	Accuracy	Precision	Recall	F1-Score
AppScanner [12]	89.54	89.84	89.68	88.92
CUMUL [29]	56.75	61.71	57.38	55.13
BIND [13]	84.57	86.81	83.82	83.96
FlowPrint [7]	81.46	65.34	70.02	65.73
DF [14]	77.87	78.86	78.19	75.93
FS-Net [15]	88.46	88.46	89.20	88.40
GraphDApp [31]	87.89	82.26	82.60	82.34
TSCRNN [17]	N/A	98.70	98.60	98.70
Deeppacket [16]	96.40	96.50	96.31	96.41
wang [5]	99.17	99.20	99.23	99.21
PERT [22]	99.09	99.11	99.10	99.11
ET-BERT (packet) [4]	99.15	99.16	99.16	99.16
ET-BERT (flow) [4]	99.29	99.30	99.30	99.30
BFCN [9]	99.39	99.41	99.40	99.40
Proposed	99.49	99.51	99.50	99.50

**Table 5:** Performance for each type of malicious traffic classification.

		ET-BERT [4]			Proposed			Wang [5]	
**Class**	**Precision**	**Recall**	**F1-Score**	**Precision**	**Recall**	**F1-Score**	**Precision**	**Recall**	**F1-Score**
BitTorrent	100	100	100	100	100	100	100	100	100
Cridex	100	100	100	100	100	100	100	100	100
Facetime	100	100	100	100	100	100	100	100	100
FTP	100	100	100	100	100	100	100	100	100
Geodo	100	99.9	99.9	100	100	100	100	100	100
Gmail	98.4	99.3	98.8	98	100	99.0	98	100	99
Htbot	99.8	100	99.9	100	100	100	100	100	100
Miuref	100	100	100	100	100	100	100	100	100
MySQL	100	100	100	100	100	100	100	100	100
Neris	96.3	92.9	94.6	97.9	94.0	95.9	94	94	94
Nsis-ay	99.8	99.0	99.4	100	98.0	99.0	100	98	99
Outlook	99.2	98.1	98.7	100	100	100	100	100	100
Shifu	99.9	99.9	99.9	100	100	100	100	100	100
Skype	99.8	100	99.9	100	100	100	100	100	100
SMB	100	100	100	100	100	100	100	100	100
Tinba	100	100	100	100	100	100	100	100	100
Virut	90.7	95.6	93.1	94.3	100	97.1	94.1	96	95
Warcraft	100	99.9	99.9	100	100	100	100	100	100
Weibot	100	100	100	100	100	100	100	98	99
Zeus	100	100	100	100	100	100	100	100	100

## Data Availability

Publicly available datasets were analyzed in this study. This data can be found here: https://github.com/yungshenglu/USTC-TFC2016.
